# Differences in physical activity in subjects with psychosis versus a control group

**DOI:** 10.1192/j.eurpsy.2022.1970

**Published:** 2022-09-01

**Authors:** A.L. Montejo, B. Buch, M.J. López, M.T. Arias, M.D. Corrales, E. Dominguez, C. Matos, B. Cortés, Y. Santana, I. Valrriberas, J. Matías, T. Prieto, M. Gómez-Marcos, L. García-Ortiz, J.M. Acosta

**Affiliations:** 1 University of Salamanca, Psychiatry, Salamanca, Spain; 2 IBSAL, Neurociencias, Salamanca, Spain; 3 Hospital universitario, Psiquiatría, salamanca, Spain; 4 hospital universitario, Psiquiatría, salamanca, Spain; 5 University of Salamanca, Atencion Primaria, Salamanca, Spain

**Keywords:** schizophrénia, Physical exercize, Psychosis, physical health

## Abstract

**Introduction:**

Psychiatric illnesses are related with a reduced life expectancy and an increase of mortality rates (around 60%) mainly associated with cardiovascular diseases [1]. The high prevalence of obesity, metabolic syndrome, diabetes mellitus and tobacco use among these patients undoubtelly predispose to the impairment in physical health and mortaility increase. Regular physical activity in the general population is associated with a decrease in cardiovascular risk but litle is know about iss influence in some chronic and severe mental disorders like schizophrenia ^[2]^.

**Objectives:**

To quantify the physical activity performed by a sample of subjects with psychosis, borth males and female, compared to a control group.

**Methods:**

A sample composed of 141 patients with schizoprenia was compared to 103 healthy subjects as a control group. The International Physical Activity Questionnaire - Short Form (IPAQ) scale was applied to all participants. The time (minutes) of physical activity performed in a week (METs) was collected by each participant ^[3]^.

**Results:**

The differences in the total physical activity Mets for the patients with schizophrenia were highly significant (p = 0.001), showing a lower degree of physical activity compared to the control group. A higher and significant percentage of sedentary lifestyle among the psychiatric group (64.5%), compared to 35.5% in the control group was found.

**Conclusions:**

The group of pateints with Schizophrenia showed a significant higher sedentary lifestile including less physical activity. This finding could be highly related with a higher risk of cardiovascular disease and deterioration of the physical health.

**Disclosure:**

No significant relationships.

